# Grip Strength Decline and Its Determinants in the Very Old: Longitudinal Findings from the Newcastle 85+ Study

**DOI:** 10.1371/journal.pone.0163183

**Published:** 2016-09-16

**Authors:** Antoneta Granic, Karen Davies, Carol Jagger, Thomas B. L. Kirkwood, Holly E. Syddall, Avan A. Sayer

**Affiliations:** 1 Institute of Neuroscience, Newcastle University, Newcastle upon Tyne, United Kingdom; 2 NIHR Newcastle Biomedical Research Centre in Ageing and Chronic Disease, Newcastle University, and Newcastle upon Tyne NHS Foundation Trust, Newcastle upon Tyne, United Kingdom; 3 Newcastle University Institute for Ageing, Campus for Ageing and Vitality, Newcastle upon Tyne, United Kingdom; 4 Institute of Health and Society, Newcastle University, Baddiley-Clark, Richardson Road, Newcastle upon Tyne, United Kingdom; 5 Institute for Cell and Molecular Biosciences, Newcastle University, Newcastle upon Tyne, United Kingdom; 6 MRC Lifecourse Epidemiology Unit, University of Southampton, Southampton, United Kingdom; 7 NIHR Collaboration for Leadership in Applied Health Research and Care, Wessex, University of Southampton, Southampton, United Kingdom; University of Sydney, AUSTRALIA

## Abstract

**Background:**

Weak grip strength (GS) is a key component of sarcopenia and frailty and a powerful predictor of mortality, morbidity and disability. Despite increasing interest in understanding GS across the lifespan, little is known about GS decline in the very old (aged ≥85). We examined trajectories of GS in very old adults and identified the determinants.

**Methods:**

GS (kg) was measured four times over 5 years in 319 men and 526 women participating in the Newcastle 85+ Study. A weak GS sub-cohort was identified as having strength of ≤27 kg (men), and ≤16 kg (women) at baseline and follow-up. Mixed models were used to establish trajectories of GS and associated factors in all participants, men and women, and in those with weak GS.

**Results:**

Men’s mean grip strength was 24.42 (SD = 6.77) kg, and women’s 13.23 (4.42) kg (p<0.001) at baseline, with mean absolute change of -5.27 (4.90) kg and -3.14 (3.41), respectively (p<0.001) by 5-year follow-up. In the time-only mixed model, men experienced linear annual decline in GS of -1.13 (0.8) kg (β (SE), p<0.001), whilst women’s decline although slower, accelerated by -0.06 (0.02) kg (p = 0.01) over time. In the saturated model, higher baseline physical activity, height, fat-free mass, better self-rated health, and not having arthritis in hand(s) were associated with stronger GS initially in both sexes. Annual GS decline in men and participants with weak GS who were highly physically active was slower by 0.95 and 0.52 kg, respectively compared with inactive counterparts.

**Conclusion:**

Grip strength decline in the very old followed linear (men) and curvilinear (women) trends. High levels of physical activity were protective of GS loss in men (but not in women) and in those with weak GS. Thus maintaining muscle strength in later life is important to reduce the morbidity and mortality in the very old.

## Introduction

Grip strength (GS) is recognised as an objective measure of upper-body and general muscle strength [1] and a key component of the geriatric syndromes of sarcopenia [2] and frailty [3]. A number of studies have confirmed age- and sex-dependent decline in GS after a peak at the age of 30 [4–14], but less is known about GS trajectories in the very old (aged ≥85). Few studies have focused on this age group alone [15–17]—despite the very old being the most vulnerable to the adverse consequences of loss of muscle strength and function [3,18]. These include increased risk of disability [19], cognitive impairment [20], depression [21], frailty [3,22], mobility decline [23], falls [22], hospitalisation [24], institutionalisation [25] and mortality [26,27] reported in younger old. GS has therefore been proposed as a biomarker of ageing [28] although there is limited evidence about the prognostic value of GS in very old adults [27,29].

To establish population norms and explore factors influencing age-related changes in GS, a number of cross-sectional and prospective cohort studies have shown stronger hand grip in men and faster decline of GS compared with women after the age of 50 [4–18]—although only a few studies were able to follow participants into very old age [5,13,16,18] For example, recent cross-sectional evaluations of harmonized data from eight UK cohorts (the Healthy Ageing across the Life Course) of individuals aged 50 to ≥90 have shown stronger GS in men and age-dependent decline, but narrowing of the sex differences in GS in very advanced age [10]—a trend also observed in other British cohort [7] and international population-based studies [5,11,13,14]. A lifespan approach applied to GS data from 12 British cohorts (ages 4 to 90) revealed a steady decline in GS from midlife onwards, and a sharp increase in weak GS prevalence in late life, defined as strength at least 2.5 standard deviations (SD) below sex-specific peak mean (or ≤27 kg in men, and ≤16 kg in women) [4]. However, the prevalence of weak versus normal GS in the very old has not been investigated separately.

Aside from age and sex, previous studies exploring factors influencing GS over time have recognized a positive association with height [1,17], BMI [8,12] (mostly in men), appendicular lean mass [30], absence of depressive symptoms [11], physical activity [31,32], self-rated health [33], and a negative association with disease burden [34], body fat [35], smoking and alcohol [14], and the risk of death [9,17], but the likely factors associated with GS trajectories in the very old are less clear [16].

We hypothesized that very old men and women, and those with weak GS may experience different GS trajectories influenced by diverse risk factors of GS decline. Utilising data from the Newcastle 85+ Study, we aimed to: (a) describe the change in GS over 5 years, and (b) identify determinants of initial level and rate of change in GS in very old men and women, and in those with weak GS at baseline and follow-up.

## Methods

### Study population

845 participants (319 men (37.8%) and 526 (62.2%) women) were examined from the Newcastle 85+ Study, a longitudinal cohort study of over 1,000 older adults born in 1921 (aged around 85 at baseline in 2006/2007), and residing in Newcastle and North Tyneside, UK. Study protocols, recruitment profile, representativeness of general population in England and Wales, and retention rate over 5-year follow-up have been described [36–38]. The study evaluated a range of bio-psycho-social factors related to health and functioning in very late life through multidimensional health assessment including measurement of GS and general practice records review. Participants were followed-up at 1.5 (wave 2), 3 (wave 3), and 5 years (wave 4). Complete data (from health assessment and medical records) were available for 845 participants at baseline. Of those, 821 (97.4%) attempted GS measurement, followed by 611, 460, and 307 participants at wave 2, 3 and 4, respectively (S1 Fig).

The study was approved by the Newcastle & North Tyneside Local Research Ethics Committee [36]. Signed informed consent was obtained from each participant, for those lacking capacity opinion was sought from their consultee (usually a relative).

### Grip strength measurement

Isometric GS [39] was measured in kilograms (kg) using a Takei hand dynamometer (Model A5401 digital 0-100kg x 0.1kd LCD) (Takei Scientific Instruments Co., Ltd., Niigata City, Japan). The standardised measurement protocol involved a standing position with elbow extension to allow the arm to hang down by the side. Participants were instructed to squeeze the dynamometer as hard as possible to assess the maximal force for each hand. Four measurements alternating between dominant and non-dominant hand were recorded, and the mean (M, SD) of four trials was calculated as a measure of overall GS and used in analyses [40].

### Potential covariates

In multivariable analyses, the following covariates identified in the literature were considered in relation to GS. Sociodemographic factors included sex, education (0–9 / 1–11 / ≥12 years), occupational class coded to the National Statistics Socio-economic Classification system (routine or manual / intermediate / higher managerial or administrative) [41], and marital status (single / widowed, separated, or divorced / married). Lifestyle factors included self-reported physical activity (low (score 0–1) / medium (score 2–6) / high (score 7–18)) [42], smoking (never / current smoker / former smoker), and current alcohol intake (yes / no). Anthropometry included height (cm) calculated from demi-span equations, weight (kg), BMI (<18.5 (underweight) / >18.5<25 (normal) / >25<30 (overweight) / 30 (obese); calculated as kg weight/m^2^ height), fat mass (FM) (kg), fat-free mass (FFM) (kg) [43], and waist-hip ratio (continuous). Health-related factors were self-rated health compared to others of the same age (excellent or very good / good / fair or poor) [37], scoring <15 points on Standardized Mini-Mental State Examination (SMMSE) (yes / no) [37], and depressive symptoms (none (0–5 points) / mild or moderate (6–7 points) / severe (8–15 points)) assessed by Geriatric Depression Scale (GDS-15) [37]. Disease burden, total number of medications (continuous), and the intake of non-prescribed medication (yes / no) were established from medical records. Diseases were grouped into arthritis, hypertension, cardiac disease, respiratory disease, cerebrovascular disease, diabetes, and cancer [37]. Having difficulty related to arthritis in hand(s) (left, right, or both, in one or more joints) during GS measurement was coded as yes / no. Total medications were also categorised (0–2 / 3–4 / ≥5) to test for polypharmacy. A retention variable (completed the study / dropped out) accounted for loss to follow-up (due to death and withdrawal) over 5 years [35].

All covariates used in multilevel analysis were baseline, fixed independent variables. Self-reported physical activity was evaluated with a purpose-designed questionnaire validated in a sub-cohort of the very old. Physical activity scores were derived from the frequency and intensity of physical activities per week, and correlated highly with actigraphy [42]. FM and FFM were estimated from inbuilt precision equation of the Tanita-305 body-fat bioimpedance instrument (Tanita Corp., Tokyo, Japan) [43]. Weak GS was defined as a strength of T-score equal or less than 2.5 below sex-specific peak mean at age of 32 (≤27 kg in men, and ≤16 kg in women) [4]. Description of selected variables has been published previously [36, 37], and further details from the study questionnaire can be found on the study’s website (http://research.ncl.ac.uk/85plus/).

### Statistical analysis

#### Unadjusted grip strength in men and women

The differences in unadjusted GS (kg) between men and women at each wave were compared using Student’s t-test, and the difference in GS across the waves were determined using repeated-measures ANOVA with Huynh-Feldt correction (Table 1).

**Table 1:** Grip strength measurements by sex in very old adults across four waves.

Wave	All	Men	Women	p*
**Baseline**, n	813	313	500	
M (SD), kg	17.54 (7.70)	24.42 (6.77)	13.23 (4.42)	<0.001
**Wave 2** (1.5-year follow-up), n	605	229	376	
M (SD), kg	16.94 (7.81)	23.77 (7.21)	12.78 (4.54)	
**Wave 3** (3-year follow-up), n	452	167	285	
M (SD), kg	16.39 (7.28)	22.68 (7.01)	12.71 (4.30)	<0.001
**Wave 4** (5-year follow-up), n	294	106	188	
M (SD), kg	14.94 (7.00)	21.05 (6.92)	11.50 (4.10)	<0.001
p^†^	<0.001	<0.001	<0.001	
**5-year mean absolute change**^‡^, n	291	106	185	
M (SD), kg	-3.92 (4.14)	-5.27 (4.90)	-3.14 (3.41)	<0.001
**5-year mean relative change**^¶^, n	291	106	185	
M (SD), %	-20.1 (23.7)	-20.0 (20.0)	-20.2 (25.6)	0.95

GS, grip strength; M, mean; SD, standard deviation.

*Student t-test.

^†^Repeated-measures ANOVA with Huynh-Feldt correction.

^‡^Calculated as: GS_wave4_ –GS_baseline_.

^¶^Calculated as: (GS_wave4_ –GS_baseline_) / GS_baseline_ * 100%.

#### Trajectory of grip strength across four waves

GS data were normally distributed at each assessment (wave) and used as continuous dependent variable. We fitted liner mixed models to examine (a) the trajectory in GS over 5 years, and (b) factors associated with initial level and rate of annual change in GS in the entire cohort (i.e. those with a complete GS measure at baseline; n = 813), and separately by sex, and in those with weak versus normal GS at baseline and follow-up (thereafter weak and normal GS sub-cohort). The model specifications and building strategy are described in the S1 Methods. Linear mixed (multilevel) models take into account both individual trajectories of change (intra-individual variability at Level 1; random effects) and population averages (inter-individual variability at Level 2; fixed effect) by using all available measurement and including participants with incomplete data [44]. The Level 1 sub-model evaluated how GS changed over time for each participant. The Level 2 sub-model examined the effect of covariates on GS change.

Time was scaled in years (continuous) to account for both linear (i.e. constant rate of change over 5 years) and quadratic (i.e. acceleration or deceleration in rate of change) GS trajectory. All predictors were fixed (baseline), and continuous variables (height and FFM) were centred to sex-specific mean to aid the interpretability of intercepts and slopes. Negative β estimates (coefficients) indicated weaker GS and positive described stronger GS compared to the specified referent group. β coefficients for predictors (covariates) represented their effect on the GS intercept. The coefficient for Time represented average annual linear change, and Time*Time captured additional quadratic (curvilinear) change in GS. A significant coefficient of time interactions with a covariate indicated whether time slopes varied by the covariate (Tables 2 and 3). All models included a random intercept and slope (i.e. taking into account each participant’s GS at baseline and the linear slope).

**Table 2:** Grip strength trajectory estimates^†^ in the entire cohort, men and women over 5 years.

Fixed effects^‡^	Entire cohort				Men				Women			
	n = 813				n = 313				n = 500			
	Model 1^¶^		Model 2^#^		Model 1		Model 2		Model 1		Model 2	
	β (SE)	p	β (SE)	p	β (SE)	p	β (SE)	p	β (SE)	p	β (SE)	p
**GS intercept**	17.44 (0.27)	<0.001	7.13 (0.97)	<0.001	24.47 (0.39)	<0.001	14.54 (2.05)	<0.001	13.14 (0.20)	<0.001	13.78 (2.47)	<0.001
Sex												
men			10.81 (0.37)	<0.001								
women (ref)			0									
Physical activity												
high			4.06 (0.53)	<0.001			5.00 (1.02)	<0.001			2.74 (0.54)	<0.001
medium			2.69 (0.49)	<0.001			3.57 (1.02)	0.001			1.58 (0.48)	0.001
low (ref)			0									
Height			0.23 (0.04)	<0.001			0.23 (0.08)	0.004			0.16 (0.04)	<0.001
FFM			0.11 (0.03)	<0.001			0.17 (0.06)	0.008			0.08 (0.03)	0.004
Waist-hip ratio											-5.77 (2.66)	0.03
Self-rated health												
excellent/ very good			1.49 (0.48)	0.002			2.15 (1.01)	0.04			1.02 (0.46)	0.003
good			0.95 (0.47)	0.05			1.08 (1.0)	0.28			0.90 (0.46)	0.05
fair/			0				0				0	
poor (ref)												
Disease count			-0.24 (0.14)	0.09							-0.34 (0.14)	0.02
Arthritis in hand(s)												
no			3.58 (0.69)	<0.001			6.18 (1.90)	0.001			2.93 (0.63)	<0.001
yes (ref)			0				0				0	
Retention												
completed the study			0.91 (0.36)	0.01							0.83 (0.35)	0.02
dropped out (ref)			0									
**GS decline**												
Time	-0.56 (0.1)	<0.001	-0.45 (0.11)	<0.001	-1.13 (0.8)	<0.001	-2.00 (0.27)	<0.001	-0.31 (0.11)	0.006	-0.43 (0.12)	<0.001
Time*Time	-0.05 (0.02)	<0.001	-0.04 (0.02)	0.04					-0.06 (0.02)	0.01	-0.05 (0.02)	0.05
**GS rate of decline (slope)**												
Time*sex												
Time*men			-0.52 (0.09)	<0.001								
Time*women (ref)			0									
Time*physical activity												
Time*high							0.95 (0.29)	0.001				
Time*medium							0.82 (0.31)	0.009				
Time*low (ref)							0					

FFM, fat-free mass; GS, grip strength; ref, reference; SE, standard error.

^†^Estimated β coefficients (SE) using GS longitudinal data.

^‡^Fixed effects assed population average change in GS. Fixed effect covariates estimated initial level and trajectory differences in GS as a function of included covariate. Random effects included both intercepts and slopes (linear change). Time in years (continuous) since the baseline interview tested linear change. Time*Time (continuous) represented curvilinear change.

^¶^Model 1 includes a linear and quadratic trend of time (except in men).

^#^Model 2 is adjusted for sociodemographic, lifestyle, anthropometry, health-related factors, retention variable, and time interaction terms. Only significant predictors and interactions at first entry were retained in the model.

**Table 3:** Grip strength trajectory estimates^†^ in the weak and normal grip strength sub-cohorts over 5 Years.

Fixed effects^‡^	Weak GS sub-cohort				Normal GS sub-cohort			
	Model 1^¶^		Model 2^#^		Model 1		Model 2	
	β (SE)	p	β (SE)	p	β (SE)	p	β (SE)	p
**GS intercept**	15.13 (0.24)	<0.001	8.41 (0.85)	<0.001	24.32 (0.42)	<0.001	15.68 (0.83)	<0.001
Sex								
men			9.28 (0.30)	<0.001			12.93 (0.42)	<0.001
women (ref)			0				0	
Marital status								
single							0.77 (0.69)	0.26
widowed/separated/divorced							0.98 (0.40)	0.02
married (ref)							0	
Occupational class								
routine/manual			-0.57 (0.29)	0.052				
intermediate			-0.16 (0.41)	0.70				
managerial/administrative (ref)			0					
								
Physical activity								
high			2.53 (0.42)	<0.001			2.09 (0.73)	0.004
medium			1.40 (0.38)	<0.001			1.56 (0.72)	0.03
low (ref)			0				0	
Height			0.08 (0.03)	0.005			0.16 (0.04)	<0.001
BMI								
underweight			-1.82 (0.65)	0.005				
normal			-0.91 (0.47)	0.052				
overweight			-0.95 (0.49)	0.053				
obese (ref)			0					
Self-rated health								
excellent/very good			0.88 (0.36)	0.02				
good			0.67 (0.35)	0.06				
fair/poor (ref)			0					
GDS								
severe							-2.22 (0.73)	0.003
mild/moderate							-0.61 (0.72)	0.4
no depression							0	
Arthritis in hand(s)								
no							3.07 (0.51)	<0.001
yes (ref)							0	
Retention								
completed the study			0.64 (0.29)	0.03				
dropped out (ref)			0					
**GS decline**								
Time	-0.37 (0.12)	0.002	-0.64 (0.18)	0.001	-0.48 (0.06)	<0.001	-0.27 (0.08)	0.001
Time*Time	-0.05 (0.02)	0.03	-0.07 (0.02)	0.005				
**GS rate of decline (slope)**								
Time*sex								
Time*men			-0.40 (0.11)	<0.001			-0.34 (0.12)	0.004
Time*women (ref)			0				0	
Time*physical activity								
Time*high			0.52 (0.17)	0.003				
Time*medium			0.56 (0.17)	0.001				
Time*low (ref)			0					

FFM, fat-free mass; GS, grip strength; ref, reference; SE, standard error.

^†^Estimated β coefficients (SE) using GS longitudinal data.

^‡^Fixed effects assed population average change in GS. Fixed effect covariates estimated initial level and trajectory differences in GS as a function of included covariate. Random effects included both intercepts and slopes (linear change). Time in years (continuous) since the baseline interview tested linear change. Time*Time (continuous) represented curvilinear change. Cut-offs of ≤27 kg (men) and ≤16 kg (women) were used to identify participants with weak versus normal GS at each assessment (1607 weak and 557 normal GS observations).

^¶^Model 1 includes a linear and quadratic trend of time (except in normal GS sub-cohort).

^#^Model 2 is adjusted for sociodemographic, lifestyle, anthropometry, health-related factors, retention variable, and time interaction terms. Only significant predictors and interactions at first entry were retained in the model.

#### Sensitivity analysis

The prevalence of weak GS (%) was calculated for each wave in the entire cohort, and separately by sex, and compared using χ^2^ test at each wave, and Cochran’s Q-test to assess differences in prevalence across waves (S1 Table). We used the cut-offs of ≤27 kg (men) and ≤16 kg (women) at each GS assessment (wave) to create a time-dependent variable and identify the weak (1607 GS observations) and normal GS sub-cohort (557 GS observations) in linear mixed models.

Sociodemographic, lifestyle, anthropometry and health-related characteristics of participants with weak versus normal GS at baseline were summarised by descriptive statistics, and compared using χ^2^ test for categorical, Student’s t-test for continuous normally distributed and Mann-Whitney U-test for non-normally distributed and ordinal variables (S2 Table). We used similar statistics to compare the characteristics of 813 participants who had a complete GS measure at baseline with those who did not (n = 32).

To account for survivor bias, mixed models were repeated in participant who completed the 5-year follow-up (thereafter ‘survivor sub-cohort’) (S3 Table).

All analyses were performed using IBM SPSS (V2.1; IBM Corporation, Armonk, NY, USA), and all statistics were reported at two-tailed α = 0.05.

## Results

### Unadjusted grip strength in men and women

Table 1 summarises unadjusted (raw) mean GS values in the entire cohort and by sex across four waves (over 5 years). At baseline, 313 men (38.5%) and 500 women (61.5%) had a complete grip GS (average of 2 measurement per each hand). As expected, men had higher mean GS initially (M (SD) = 24.42 (6.77) kg) compared with women (13.23 (4.42) kg) and thereafter (p for all <0.001), and experienced greater mean absolute change (-5.27 (4.90) kg versus -3.14 (3.41) kg (p<0.001) in women). However the mean relative change was similar in both sexes.

### Trajectory of grip strength across four waves

#### Entire cohort

The unconditional time-only model (Model 1, Table 2), showed both linear and quadratic effects of decline in GS in the entire cohort or an overall loss of -0.56 kg per year with accelerated loss of -0.05 kg annually over the 5-year follow-up (both p<0.001). In the saturated model (Model 2, Table 2), we found a significant association between GS initially and sex, physical activity, height, FFM, self-rated health, presence of arthritis in hand(s), and retention, and a significant interaction between linear time and sex, indicating a steeper rate of GS decline in men (β (SE) = -0.52 (0.09), p<0.001).

#### Men and women

In the time-only model (Model 1, Table 2), men’s GS trajectory showed a higher intercept (24.47 (0.39) kg, p<0.001) and linear decline (-1.13 (0.8) kg, p<0.001), whilst women started with lower intercept (13.14 (0.20), p<0.001) but experienced accelerated loss of -0.06 (0.02) kg (p = 0.01) per each follow-up year above the loss seen in the first year. In men, significant determinants of GS initially were: physical activity (high: 5.00 (1.02), p<0.001; medium: 3.57 (1.02), p = 0.001), height (0.23 (0.08), p = 0.004), FFM (0.17 (0.06), p = 0.008), self-rated health (excellent or very good: 2.15 (1.01), p = 0.04), and arthritis in hand(s) (6.18 (1.90), p = 0.001) (Model 2, Table 2). In women, additional significant covariates of GS included greater disease burden and waist-hip ratio which predicted a weaker GS, and retention (completing the study) which was associated with a stronger GS at baseline. Of all significant covariates, the rate of GS decline was only affected by high (0.95 (0.29), p = 0.001) and medium physical activity (0.82 (0.31), p = 0.009) in men, but not in women.

Estimated 5-year trajectories in GS based on β coefficients from the saturated model (Model 2) in the entire cohort and by sex are presented in S2 Fig (panel A).

#### Weak and normal GS sub-cohorts

To account for weak baseline GS affecting the trajectory in GS in very old adults and the possibility of the floor effect in women over follow-up, we examined the association (fixed effects) between GS and important covariates (initially and over time) in participants belonging to the weak and normal GS sub-cohort (Table 3; S2 Fig, panel B). The time-only model (Model 1) revealed a linear (-0.37 (0.12), p = 0.002) and quadratic (-0.05 (0.02), p = 0.03) (acceleration) decline in GS over 5 years in those with overall weak GS (as in the entire cohort), but linear loss of GS in the normal GS sub-cohort (-0.48 (0.06), p<0.001). In both groups, sex (men), higher physical activity (medium and high), and height were significantly associated with a stronger GS initially. Additional determinants of GS in the weak GS sub-cohort included BMI (and not FFM; data not shown), self-rated health, arthritis in hand(s), and retention. In both sub-cohorts, men experienced steeper slopes of GS decline compared with women, but only those in the weak GS sub-cohort benefited from physical activity, and had slower rate of GS decline (medium: 0.52 (0.17) kg, p = 0.003); high: 0.56 (0.17), p = 0.001 compared with low physical activity).

#### Sensitivity analyses

Participants with a complete GS measure (n = 813) were more likely to be men (p = 0.02), to be more educated (p = 0.01) and physically active (p<0.001), and less likely to be cognitively impaired (p<0.001), but did not differ on any other health and anthropometry measures (details not shown).

Baseline prevalence of weak GS was higher in women (74.2% vs 63.6% in men, p<0.001), and increased over time for both sexes (p<0.001), but levelled off by wave 4 (S1 Table). Compared with participants with normal GS at baseline (n = 243), those with weak GS (n = 570) were more likely to be women (p = 0.001), to have low physical activity (p<0.001), to be shorter (p<0.001), have lower weight and be classified as underweight, to have lower FM and FFM (all p<0.001), to be cognitively impaired (SMMSE <15 points) (p = 0.002), to have greater disease burden (p = 0.005), arthritis in hand(s) (p<0.001), history of falls (p = 0.03), to take more medication (p<0.001), and not to complete the study (p<0.001) (S2 Table).

The models fitted to survivor sub-cohort (n = 343) showed similar GS trajectory and determinants of initial level and rate of change in GS (S3 Table).

## Discussion

To our knowledge, this is the first prospective cohort study to (a) quantify sex-specific decline in GS, and (b) describe determinants thereof in very old adults (aged ≥85) living in Britain. Decline of GS in the entire cohort followed a nonlinear trajectory (i.e. accelerated over 5 years), and was more pronounced in men. Men had about 11 kg stronger initial GS than women and declined linearly (-2 kg/year), whilst women’s initial rate of loss was -0.43 kg but accelerated by -0.05 kg through the follow-up. For both, higher physical activity, height, FFM, and better self-rated health were associated with a stronger grip initially. Disease burden predicted weaker GS only in women, whilst the presence arthritis in hand(s) was associated with worst GS in both sexes. Men, but not women who were physically active experienced slower rate of GS decline. Similarly, the rate of decline was steeper among men in the weak and normal GS sub-cohorts, but more physically active participants in the weak GS group experienced slower decline.

Consistent with previous research which included very old adults, the present study showed that GS declines with advancing age in both sexes, and that men experienced steeper slope of decline [7,9–11,14,18], although they started with stronger GS compared with women—confirming also that those who have greater GS initially tend to lose more and faster over time [10,14]. Women experienced accelerated (nonlinear) GS decline, which was also observed among 2,200 Danish nonagenarians followed up for 8 years [13], although previous analysis of several Danish cohorts (aged ≥70) suggested a horizontal plateau in the oldest women (aged ≥95) when withdrawal and mortality was accounted for [18]. GS in women participating in the Mini-Finland Health examination declined linearly 4 N (0.41 kg/year) over 10-year follow-up after the age of 80, and additionally by 5.2 N (0.53 kg/year) when mortality (right censoring) was taken into consideration [9]. Due to change in the rate of decline in women’s GS, the annual rate of loss in our cohort is difficult to summarize, but fully adjusted estimates indicate less steep GS decline in women compared with men. In the Leiden 85+ Study, men’s absolute loss in GS was 1.5 kg/years and women’s 0.9 kg/year over 4 years, although relative loss between sexes was similar (-19% in men and -16% in women) [29]—a trend that we confirmed in our cohort (i.e. -20% over 5 years). In addition, while women were more likely to have weak baseline GS, the prevalence of weak grip was similar between men and women 5 years later.

Observed sex differences in GS loss may be partially explained by body composition [43], multimorbidity [37,45], and survival [46]. Sex differences in muscle quality (loss of lean mass and increase in fat mas) [43] with advancing age may be one of the factors contributing to sex differences in GS decline. In our cohort, disease burden was a significant predictor of weaker GS initially in women, but not in men. Also, having difficulty with GS assessment because of arthritis in hand(s) but not polypharmacy predicted worse baseline GS in both sexes. Despite weaker GS over time, which predicted accelerated decline in disability and global cognition in the very old in the Leiden 85+ Study [16], women’s less steep GS decline adds to the previously observed male-female health-survival paradox [45,46]. In the present cohort, we have previously shown that women lived longer with more disability than men, which was attributed to the sex differences in the type of diseases and their impact on disablement process [45]. Women’s longer life expectancy (spent in less good health and with more disabilities) and the greater survival of healthier men [46] (survivor effect) in very old age may partly explain the sex differences in GS.

Although multilevel models use all available data, selection bias due to mortality and withdrawal is still a possibility. We included the variable ‘retention’ (i.e. completed the study over 5 years or not) as an inter-individual covariate to account for selective mortality/withdrawal. This method was used in the recent study of older Japanese (aged ≥65) investigating bio-psycho-social factors of GS trajectory [35], and in other prospective studies of change in health in older adults [47]. We also explored GS trajectory in survivors and confirmed the results.

The main finding from the present study points to the role of physical activity in the rate of decline in GS in men and in participants with overall weak GS. Compared with those reporting low levels of physical activity, men and participants with overall weak GS who had both high and medium levels of physical activity at baseline experienced slower loss of GS (0.95 and 0.82 kg/year in men, and 0.52 and 0.56 kg/year in the weak GS sub-cohort, respectively) after adjusting for important covariates. To our knowledge, this is the first prospective study to report the importance of physical activity for GS maintenance in very late life, including in those who had low GS over 5 years. Others have shown similar and opposite sex-specific influence of physical activity on GS in general and older adults population. For example, higher leisure time physical activity (LTPA) assessed longitudinally across midlife was associated with stronger GS at age 60–64 in both men and women [31]. In Danish Health2006 study (aged 19–72), higher LTPA was also significantly related to stronger GS in both sexes [48], whilst women (aged ≥64) in the Toledo Study of Healthy Aging benefited more from higher physical activity then men, but adults aged ≥85 benefited less compared with younger counterparts [49]. The finding from the present study suggests the importance of maintaining higher level of physical activity for muscle strength well into late adulthood.

Our study has several limitations: (a) the findings regarding predictors of the initial level and decline in GS came from observational data and may not be causal; (b) dichotomisation of certain predictors (e.g. current alcohol intake), and the fact that all predictors were baseline (fixed) covariates may have affected the results; (c) participants with a complete GS measure at baseline were more likely to be men, well-educated and more physically active compared with those not included in analyses; (d) as with any cohort of very old adults, mortality was high [38] and may have resulted in biased sample of very fit survivors, especially men; (e) although we adjusted for mortality and withdrawal and included important confounders, the results may be influenced by selective mortality and by uncontrolled confounding (e.g. biological factors, individual diseases, nutritional status); and (f) our sample was derived from a single urban area in North-East England, with predominantly white ethnicity and had a slight under-representativeness of women compared with census data from 2001. The study has several strengths: (a) the prospective design; (b) multilevel analysis of stratified sub-cohorts (by sex and presence of sex-specific weak GS at baseline and follow-ups); (c) inclusion of important confounders reported in the literature; and (d) a broad representativeness of the general population in England and Wales.

### Conclusions

We have found different GS trajectories in very old men and women, and recognized factors influencing GS decline in advancing age. Men and participants with overall weak GS who were physically active experienced slower rate of decline even after adjusting for important confounders. This study provided additional evidence supporting benefits of physical activity into very late life, which should be explored in other cohorts of the very old.

## Supporting Information

S1 Fig Flow chart of participants in the Newcastle 85+ Study by grip strength (GS). At baseline, 845 participants had a complete data (health assessment and medical records review). Of those, 821 participants attempted grip strength measurement (2 per each hand) and had complete multidimensional health assessment and general practice medical records review. At 5-year follow-up (wave 4), 307 remained in the study. The loss to follow-up was mainly due to mortality.
(TIF)

S2 Fig Estimated 5-year trajectories of grip strength (GS) in the entire cohort, men and women, and in the participant with weak and normal GS at baseline and follow-up in the Newcastle 85+ Study. The fully-adjusted β estimates indicated a linear decline in grip strength in men (dashed line) and curvilinear (acceleration) decline in the entire cohort (full line) and women (dotted line) over 5 years (A). On average, men lost 2 kg/year (A) and the participants in the normal grip strength sub-cohort lost -0.3 kg/year in GS (B). Women and those in the weak grip strength sub-cohort experienced accelerated rate of decline of -0.05 and -0.07 kg per each follow-up year above the average loss of -0.43 and -0.64 kg experienced in the first year, respectively.
(TIF)

S1 Methods Model building strategy. (DOCX)

S1 Table Sex-specific prevalence of weak grip strength in the Newcastle 85+ Study. (DOCX)

S2 Table Characteristics of participants with weak and normal grip strength at baseline in the Newcastle 85+ Study. (DOCX)

S3 Table Grip strength trajectory estimates in the survivor sub-cohort (n = 343) over 5 year. (DOCX)

## References

[1] Visser M, Deeg DJ, Lips P, Harris TB, Bouter LM. Skeletal muscle mass and muscle strength in relation to lower-extremity performance in older men and women. J Am Geriatr Soc 2000;48: 381–386. 10798463 10.1111/j.1532-5415.2000.tb04694.x

[2] Dam TT, Peters KW, Fragala M, Cawthon PM, Harris TB, McLean R, et al An evidence-based comparison of operational criteria for the presence of sarcopenia. J Gerontol A Biol Sci Med Sci 2014;69: 584–590. 10.1093/gerona/glu013 24737561 PMC3991139

[3] Sternberg SA, Wershof Schwartz A, Karunananthan S, Bergman H, Mark Clarfield A. The identification of frailty: a systematic literature review. J Am Geriatr Soc 2011;59: 2129–2138. 10.1111/j.1532-5415.2011.03597.x 22091630

[4] Dodds RM, Syddall HE, Cooper R, Benzeval M, Deary IJ, Dennison EM, et al Grip strength across the life course: normative data from twelve British studies. PLoS ONE 2014;9: e113637 10.1371/journal.pone.0113637 25474696 PMC4256164

[5] Seino S, Shinkai S, Fujiwara Y, Obuchi S, Yoshida H, Hirano H, et al Reference values and age and sex differences in physical performance measures for community-dwelling older Japanese: a pooled analysis of six cohort studies. PLoS ONE 2014;9: e99487 10.1371/journal.pone.0099487 24923425 PMC4055685

[6] Spruit MA, Sillen MJ, Groenen MT, Wouters EF, Franssen FM. New normative values for handgrip strength: results from the UK Biobank. J Am Med Dir Assoc 2013;14: 775.e5–11. 10.1016/j.jamda.2013.06.013 23958225

[7] Keevil VL, Hayat S, Dalzell N, Moore S, Bhaniani A, Luben R, et al The physical capability of community-based men and women from a British cohort: the European Prospective Investigation into Cancer (EPIC)-Norfolk study. BMC Geriatr 2013;13: 93 10.1186/1471-2318-13-93 24020915 PMC3846689

[8] Hardy R, Cooper R, Aihie Sayer A, Ben-Shlomo Y, Cooper C, Deary IJ, et al Body mass index, muscle strength and physical performance in older adults from eight cohort studies: the HALCyon Programme. PLoS ONE 2013;8: e56483 10.1371/journal.pone.0056483 23437142 PMC3577921

[9] Stenholm S, Härkänen T, Sainio P, Heliövaara M, Koskinen S. Long-term changes in handgrip strength in men and women—accounting the effect of right censoring due to death. J Gerontol A Biol Sci Med Sci 2012;67: 1068–1074. 10.1093/gerona/gls064 22421705

[10] Cooper R, Hardy R, Aihie Sayer A, Ben-Shlomo Y, Birnie K, Cooper C, et al Age and gender differences in physical capability levels from mid-life onwards: the harmonisation and meta-analysis of data from eight UK cohort studies. PLoS ONE 2011;6: e27899 10.1371/journal.pone.0027899 22114723 PMC3218057

[11] Ishizaki T, Furuna T, Yoshida Y, Iwasa H, Shimada H, Yoshida H, et al Declines in physical performance by sex and age among nondisabled community-dwelling older Japanese during a 6-year period. J Epidemiol 2011;21: 176–183. 21368451 10.2188/jea.JE20100138 PMC3899406

[12] Massy-Westropp NM, Gill TK, Taylor AW, Bohannon RW, Hill CL. Hand grip strength: age and gender stratified normative data in a population-based study. BMC Res Notes 2011;4: 127 10.1186/1756-0500-4-127 21492469 PMC3101655

[13] Oksuzyan A, Maier H, McGue M, Vaupel JW, Christensen K. Sex differences in the level and rate of change of physical function and grip strength in the Danish 1905-Cohort Study. J Aging Health 2010;22: 589–610. 10.1177/0898264310366752 20453156 PMC2952838

[14] Nahhas RW, Choh AC, Lee M, Chumlea WM, Duren DL, Siervogel RM, et al Bayesian longitudinal plateau model of adult grip strength. Am J Hum Biol 2010;22: 648–656. 10.1002/ajhb.21057 20737612 PMC3988672

[15] Legrand D, Adriaensen W, Vaes B, Matheï C, Wallemacq P, Degryse J. The relationship between grip strength and muscle mass (MM), inflammatory biomarkers and physical performance in community-dwelling very old persons. Arch Gerontol Geriatr 2013;57: 345–351. 23830056 10.1016/j.archger.2013.06.003

[16] Taekema DG, Gussekloo J, Maier AB, Westendorp RG, de Craen AJ. Handgrip strength as a predictor of functional, psychological and social health. A prospective population-based study among the oldest old. Age Ageing 2010;39: 331–337. 10.1093/ageing/afq022 20219767

[17] Proctor DN, Fauth EB, Hoffman L, Hofer SM, McClearn GE, Berg S, et al Longitudinal changes in physical functional performance among the oldest old: insight from a study of Swedish twins. Aging Clin Exp Res 2006;18: 517–530. 17255642 10.1007/BF03324853

[18] Frederiksen H, Hjelmborg J, Mortensen J, McGue M, Vaupel JW, Christensen K. Age trajectories of grip strength: cross-sectional and longitudinal data among 8,342 Danes aged 46 to 102. Ann Epidemiol 2006;16: 554–562. 16406245 10.1016/j.annepidem.2005.10.006

[19] Rantanen T, Avlund K, Suominen H, Schroll M, Frändin K, Pertti E. Muscle strength as a predictor of onset of ADL dependence in people aged 75 years. Aging Clin Exp Res 2002;4: 10–15. 12475129

[20] Clouston SAP, Brewster P, Kuh D, Richards M, Cooper R, Hardy R, et al The dynamic relationship between physical function and cognition in longitudinal aging cohorts. Epidemiol Rev 2013;35: 33–50. 10.1093/epirev/mxs004 23349427 PMC3578448

[21] Fukumori N, Yamamoto Y, Takegami M, Yamazaki S, Onishi Y, Sekiguchi M, et al Association between hand-grip strength and depressive symptoms: Locomotive Syndrome and Health Outcomes in Aizu Cohort Study (LOHAS). Age Aging 2015;44: 592–598. 10.1093/ageing/afv013 25712514

[22] Xue QL, Walston JD, Fried LP, Beamer BA. Prediction of risk of falling, physical disability, and frailty by rate of decline in grip strength: the women's health and aging study. Arch Intern Med 2011;171: 1119–1121. 10.1001/archinternmed.2011.252 21709116

[23] Hicks GE, Shardell M, Alley DE, Miller RR, Bandinelli S, Guralnik J, et al Absolute strength and loss of strength as predictors of mobility decline in older Adults: the InCHIANTI Study. J Gerontol A Biol Sci Med Sci 2012;67A: 66–73. 10.1093/gerona/glr055 PMC3260485 21546582

[24] Kerr A, Syddall HE, Cooper C, Turner GF, Briggs RS, Sayer AA. Does admission grip strength predict length of stay in hospitalised older patients? Age Ageing 2006;35: 82–84. 16364940 10.1093/ageing/afj010

[25] Roberts HC, Syddall HE, Sparkes J, Ritchie J, Butchart J, Kerr A, et al Grip strength and its determinants among older people in different healthcare settings. Age Ageing 2014; 43: 241–246. 10.1093/ageing/aft118 23926093 PMC3918578

[26] Leong DP, Teo KK, Rangarajan S, Lopez-Jaramillo P, Avezum A Jr, Orlandini A, et al Prognostic value of grip strength: findings from the Prospective Urban Rural Epidemiology (PURE) study. Lancet 2015;386: 266–273. 10.1016/S0140-6736(14)62000-6 25982160

[27] Cevenini E, Cotichini R, Stazi MA, Toccaceli V, Palmas MG, Capri M, et al Health status and 6 years survival of 552 90+ Italian sib-ships recruited within the EU Project GEHA (GEnetics of Healthy Ageing). Age (Dordr) 2014;36: 949–966. 24323371 10.1007/s11357-013-9604-1 PMC4039258

[28] Sayer AA, Kirkwood TB. Grip strength and mortality: a biomarker of ageing? Lancet 2015;386: 226–227. 10.1016/S0140-6736(14)62349-7 25982159

[29] Ling CH, Taekema D, de Craen AJ, Gussekloo J, Westendorp RG, Maier AB. Handgrip strength and mortality in the oldest old population: the Leiden 85-plus study. CMAJ 2010;182:429–435. 10.1503/cmaj.091278 20142372 PMC2842834

[30] Cawthon PM, Peters KW, Shardell MD, McLean RR, Dam TT, Kenny AM, et al Cutpoints for low appendicular lean mass that identify older adults with clinically significant weakness. J Gerontol A Biol Sci Med Sci 2014;69: 567–575. 10.1093/gerona/glu023 24737559 PMC3991141

[31] Dodds R, Kuh D, Aihie Sayer A, Cooper R. Physical activity levels across adult life and grip strength in early old age: updating findings from a British birth cohort. Age Ageing 2013;42: 794–798. 10.1093/ageing/aft124 23981980 PMC3809720

[32] Sternäng O, Reynolds CA, Finkel D, Ernsth-Bravell M, Pedersen NL, Dahl Aslan AK. Factors associated with grip strength decline in older adults. Age Aging 2015;44: 269–274. 10.1093/ageing/afu170 PMC4400526 25362503

[33] Sayer AA, Syddall HE, Martin HJ, Dennison EM, Roberts HC, Cooper C. Is grip strength associated with health-related quality of life? Findings from the Hertfordshire Cohort Study. Age Ageing 2006;35: 409–415. 16690636 10.1093/ageing/afl024

[34] Cheung C-L, Nguyen US, Au E, Tan KC, Kung AW. Association of handgrip strength with chronic diseases and multimorbidity: a cross-sectional study. Age Ageing 2013;35: 929–941. 10.1007/s11357-012-9385-y PMC3636411 22314403

[35] Botoseneanu A, Bennett JM, Nyquist L, Shinkai S, Fujiwara Y, Yoshida H, et al Cardiometabolic risk, socio-psychological factors, and trajectory of grip strength among older Japanese adults. J Aging Health 2015;27: 1123–1146. 10.1177/0898264315577587 25903979

[36] Collerton J, Barrass K, Bond J, Eccles M, Jagger C, James O, et al The Newcastle 85+ study: biological, clinical and psychological factors associated with healthy ageing: study protocol. BMC Geriatrics 2007; 7: 14 17594470 10.1186/1471-2318-7-14 PMC1924857

[37] Collerton J, Davies K, Jagger C, Kingston A, Bond J, Eccles MP, et al Health and disease in 85 year olds: baseline findings from the Newcastle 85+ cohort study. BMJ 2009;399: b4904. 10.1136/bmj.b4904 PMC2797051 20028777

[38] Davies K, Kingston A, Robinson L, Hughes J, Hunt JM, Barker SA, et al Improving retention of very old participants in longitudinal research: experiences from the Newcastle 85+ study. PLoS ONE 2014;9: e108370 10.1371/journal.pone.0108370 25302500 PMC4193743

[39] Roberts HC, Denison HJ, Martin HJ, Patel HP, Syddall H, Cooper C, et al A review of the measurement of grip strength in clinical and epidemiological studies: towards a standardised approach. Age Ageing 2011;40: 423–429. 10.1093/ageing/afr051 21624928

[40] Haidar SG, Kumar D, Bassi RS, Deshmukh SC. Average versus maximum grip strength: Which is more consistent? J Hand Surg Br 2004;29B: 82–84. 10.1016/j.jhsb.2003.09.012 14734079

[41] Chandola T, Jenkinson C. The new UK National Statistics Socio-Economic Classification (NS-SEC); investigating social class differences in self-reported health status. J Public Health Med 2000;22: 182–190. 10912557 10.1093/pubmed/22.2.182

[42] Innerd P, Catt M, Collerton J, Davies K, Trenell M, Kirkwood TB, et al A comparison of subjective and objective measures of physical activity from the Newcastle 85+ study. Age Ageing 2015; 44: 691–694. 10.1093/ageing/afv062 26018999 PMC4476851

[43] Siervo M, Prado C, Hooper L, Munro A, Collerton J, Davies K, et al Serum osmolarity and haematocrit do not modify the association between the impedance index (Ht2/Z) and total body water in the very old: The Newcastle 85+ Study. Arch Gerontol Geriatr 2015;60: 227–232. 10.1016/j.archger.2014.09.004 25288578

[44] Singer JD, Willett JB. Applied longitudinal data analysis: Modeling change and event occurrence, 1st ed New York, NY: Oxford University Press; 2003.

[45] Kingston A, Davies K, Collerton J, Robinson L, Duncan R, Bond J, et al The contribution of diseases to the male-female disability-survival paradox in the very old: results from the Newcastle 85+ study. PLoS One 2014;9: e88016 10.1371/journal.pone.0088016 24516578 PMC3917849

[46] Oksuzyan A, Juel K, Vaupel JW, Christensen K. Men: good health and high mortality. Sex differences in health and aging. Aging Clin Exp Res 2008;20: 91–102. 18431075 10.1007/bf03324754 PMC3629373

[47] Quiñones AR, Liang J, Bennett JM, Xu X, Ye W. How does the trajectory of multimorbidity vary across Black, White, and Mexican Americans in middle and old age? J Gerontol B Psychol Sci Soc Sci 2011;66: 739–749. 10.1093/geronb/gbr106 21968384 PMC3198247

[48] Aadahl M, Beyer N, Linneberg A, Thuesen BH, Jørgensen T. Grip strength and lower limb extension power in 19–72-year-old Danish men and women: the Health2006 study. BMJ Open 2011;1: e000192 10.1136/bmjopen-2011-000192 22021882 PMC3191596

[49] Gómez-Cabello A, Carnicero JA, Alonso-Bouzón C, Tresguerres JÁ, Alfaro-Acha A, Ara I, et al Age and gender, two key factors in the associations between physical activity and strength during the ageing process. Maturitas 2014;78: 106–112. 10.1016/j.maturitas.2014.03.007 24720906

