# Supplementary figures and images for: Grip Strength Decline and Its Determinants in the Very Old: Longitudinal Findings from the Newcastle 85+ Study
Source: PLoS One. 2016 Sep 16;11(9):e0163183. doi: 10.1371/journal.pone.0163183 (PMC5026378; doi:10.1371/journal.pone.0163183)

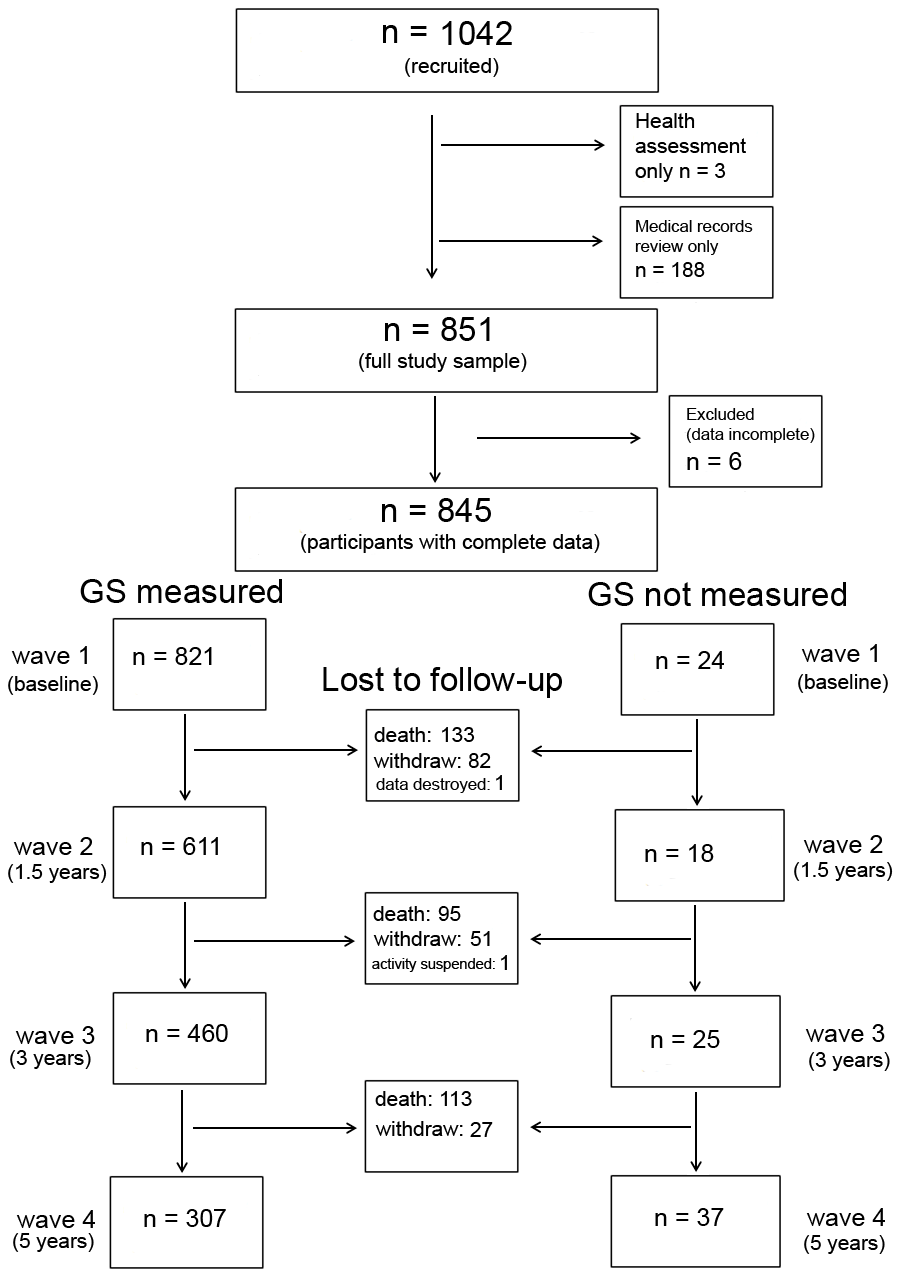

Supplement: S1 Fig — At baseline, 845 participants had a complete data (health assessment and medical records review). Of those, 821 participants attempted grip strength measurement (2 per each hand) and had complete multidimensional health assessment and general practice medical records review. At 5-year follow-up (wave 4), 307 remained in the study. The loss to follow-up was mainly due to mortality. (TIF) [file pone.0163183.s001.tif]

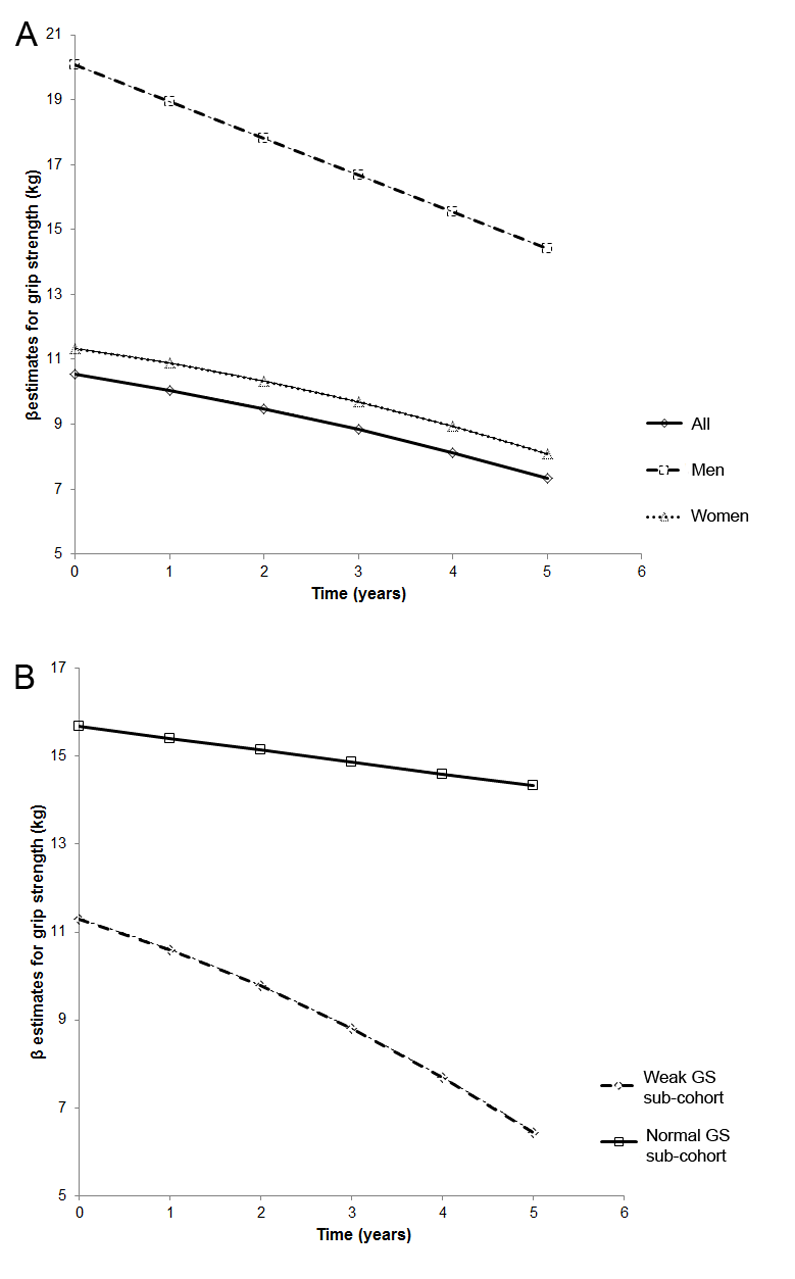

Supplement: S2 Fig — The fully-adjusted β estimates indicated a linear decline in grip strength in men (dashed line) and curvilinear (acceleration) decline in the entire cohort (full line) and women (dotted line) over 5 years (A). On average, men lost 2 kg/year (A) and the participants in the normal grip strength sub-cohort lost -0.3 kg/year in GS (B). Women and those in the weak grip strength sub-cohort experienced accelerated rate of decline of -0.05 and -0.07 kg per each follow-up year above the average loss of -0.43 and -0.64 kg experienced in the first year, respectively. (TIF) [file pone.0163183.s002.tif]
