# Supplementary material for: Grip Strength Decline and Its Determinants in the Very Old: Longitudinal Findings from the Newcastle 85+ Study
Source: PLoS One. 2016 Sep 16;11(9):e0163183. doi: 10.1371/journal.pone.0163183 (PMC5026378; doi:10.1371/journal.pone.0163183)
Supplement: S1 Methods — (DOCX) [file pone.0163183.s003.docx]

**S1 Methods**

**Model building strategy**

We used the following procedure to build growth curve models for grip strength (GS). In the first step, we used unconditional means model without Time to discern inter-individual and intra-individual variability in GS and to calculate interclass correlation coefficient (ρ)—a proportion of the total variation in GS that lay between individuals (ρ=83.9% for the entire cohort; ρ=72.6% for men, and ρ=66.1% for women; ρ=76.7% for the weak GS sub-cohort; ρ=90.5% for the normal GS sub-cohort; details not shown). In the next step we entered linear (Time) and quadratic (Time*Time) (at Level 1) to assess GS trajectory, followed by stepwise entry of sociodemographic, lifestyle, anthropometric, health-related factors and retention variable (at Level 2) to test whether initial hand grip (intercept) varied by each covariate. Retention variable (binary) was included as an inter-individual covariate to adjust for selective mortality and withdrawal^35^. For parsimony, only significant associations (α≤0.05) at first entry were retained, and β coefficients reported (Model 1 and Model 2, Table 2 and Table 3). Lastly, interaction terms between Time and significant covariates were entered to test for varying slopes (rate of change) by each individually. Only significant interaction terms were retained and reported (Model 2). Model building procedure was repeated separately in men and women, and in the sub-cohorts with weak and normal GS.

Model fit of two nested models were compared by inspecting Akaike’s Information Criterion, and computing χ^2^ likelihood-ratio test with the degrees of freedom set as the difference in the number of parameters between the two models. Based on described model specification, intercept estimated GS at baseline for an individual with 0 value on all significant predictors retained in the model, and the linear and quadratic coefficient represented change (i.e. decrease or loss) in GS per year since baseline for that individual. Predictor codes are presented in S2 Table.

All parameters (β coefficients) were estimated using restricted maximum likelihood, and scaled identity and unstructured covariance matrix at Level 1 and Level 2, respectively (SPSS MIXED procedure).
