# Supplementary material for: Grip Strength Decline and Its Determinants in the Very Old: Longitudinal Findings from the Newcastle 85+ Study
Source: PLoS One. 2016 Sep 16;11(9):e0163183. doi: 10.1371/journal.pone.0163183 (PMC5026378; doi:10.1371/journal.pone.0163183)
Supplement: S1 Table — (DOCX) [file pone.0163183.s004.docx]

**S1 Table.** Sex-specific prevalence of weak grip strength in the Newcastle 85+ Study^*^

| Wave | All | Men | Women | p† |
| --- | --- | --- | --- | --- |
| **Baseline** |  |  |  |  |
| yes % (n) | 70.1 (570) | 63.6 (119) | 74.2 (371) | <0.001 |
| no 5 (n) | 29.9 (243) | 36.4 (114) | 25.8 (129) |  |
| **Wave 2 (1.5-year follow-up)** |  |  |  |  |
| yes % (n) | 73.2 (144) | 67.2 (154) | 77.1 (290) | 0.008 |
| no % (n) | 26.8 (162) | 32.8 (75) | 22.9 *86) |  |
| **Wave 3** **(3-year follow-up)** |  |  |  |  |
| yes % (n) | 76.8 (347) | 72.5 (121) | 79.3 (226) | 0.1 |
| no % (n) | 23.2 (105) | 27.5 (46) | 20.7 (59) |  |
| **Wave 4 (5-year follow-up)** |  |  |  |  |
| yes % (n) | 83.7 (246) | 78.3 (83) | 86.7 (163) | 0.06 |
| no % (n) | 16.3 (48) | 21.7 (23) | 13.3 (25) |  |
| p^‡^ | <0.001 | <0.001 | <0.001 |  |

^*^Sex-specific weak grip strength was defined as performing equal to or less than 2.5 SD below young adult peak mean (≤27 kg in men and ≤16 kg in women) [4].

^†^χ^2^ test at α=0.05.

^‡^Cochran’s Q test for binary variable comparison across waves.
