# Supplementary material for: Grip Strength Decline and Its Determinants in the Very Old: Longitudinal Findings from the Newcastle 85+ Study
Source: PLoS One. 2016 Sep 16;11(9):e0163183. doi: 10.1371/journal.pone.0163183 (PMC5026378; doi:10.1371/journal.pone.0163183)
Supplement: S2 Table — (DOCX) [file pone.0163183.s005.docx]

**S2 Table.** Characteristics of participants with weak^*^ and normal grip strength at baseline in the Newcastle 85+ Study

| Characteristic | Coding | Weak grip strength | Normal grip strength | p^†^ |
| --- | --- | --- | --- | --- |
|  |  | n=570 | n=243 |  |
| **Sociodemographic** |  |  |  |  |
| Sex, % (n) |  |  |  | 0.001 |
| Women | 1 (ref) | 65.1 (371) | 53.1 (129) |  |
| Men | 0 | 34.9 (199) | 46.9 (114) |  |
| Education, % (n) |  |  |  | 0.53 |
| 0-9 years | 0 | 64.4 (362) | 62.1 (151) |  |
| 10-11 years | 1 | 22.8 (128) | 23.9 (58) |  |
| 12-20 years | 2 (ref) | 12.8 (72) | 14.0 (34) |  |
| Occupational class, % (n) |  |  |  | 0.04 |
| routine/manual | 0 | 53.1 (287) | 47.5 (112) |  |
| intermediate | 1 | 15.54 (83) | 11.9 (28) |  |
| higher managerial/ | 2 (ref) | 31.5 (170) | 40.7 (98) |  |
| administrative |  |  |  |  |
| Marital status, % (n) |  |  |  | 0.99 |
| single | 0 | 7.9 (45) | 8.2 (20) |  |
| widowed/divorced | 1 | 61.6 (350) | 61.3 (149) |  |
| married | 2 (ref) | 30.5 (173) | 30.5 (74) |  |
| **Lifestyle** |  |  |  |  |
| Physical activity, % (n) |  |  |  | <0.001 |
| low | 0 | 27.6 (151) | 8.5 (20) |  |
| medium | 1 | 44.0 (241) | 41.9 (99) |  |
| high | 2 (ref) | 28.5 (156) | 49.9 (117) |  |
| Smoking status, % (n) |  |  |  | 0.5 |
| never | 2 (ref) | 36.8 (209) | 32.5 (79) |  |
| current | 1 | 5.6 (32) | 5.8 (14) |  |
| former | 0 | 57.6 (327) | 61.7 (190) |  |
| Current alcohol intake, % (n) |  |  |  | 0.07 |
| yes | 1 (ref) | 58.6 (325) | 65.5 (156) |  |
| no | 0 | 41.4 (230) | 34.5 (82) |  |
| **Anthropometry** |  |  |  |  |
| Height (cm), M (SD) |  | 159.95 (7.57) | 164.14 (7.66) | <0.001 |
| Weight (kg), M (SD) |  | 62.09 (12.33) | 68.99 (12.92) | <0.001 |
| BMI, % (n) |  |  |  | <0.001 |
| <18.5 (underweight) | 0 | 8.6 (43) | 1.3 (3) |  |
| >18.5<25 (normal) | 1 | 53.1 (266) | 47.0 (110) |  |
| >25<30 (overweight) | 2 | 29.9 (150) | 38.5 (90) |  |
| >30 (obese) | 3 (ref) | 8.4 (42) | 13.2 (31) |  |
| Fat mass (kg), M (SD) |  | 17.99 (7.72) | 20.80 (7.51) | <0.001 |
| Fat free mas (kg), M (SD) |  | 44.0 (8.51) | 47.84 (9.46) | <0.001 |
| Waist-hip ratio, M (SD) |  | 0.88 (0.07) | 0.89 (0.08) | 0.55 |
| **Health-related factors** |  |  |  |  |
| Self-rated health, % (n) |  |  |  | <0.001 |
| excellent/very good | 0 | 36.7 (206) | 48.3 (117) |  |
| good | 1 | 37.2 (209) | 39.7 (96) |  |
| fair/poor | 2 (ref) | 26.2 (147) | 12.0 (29) |  |
| SMMSE <15 points, % (n) |  |  |  | 0.002 |
| yes | 1 (ref) | 5.4 (31) | 0.8 (2) |  |
| no | 0 | 94.6 (538) | 99.2 (241) |  |
| GDS, % (n) |  |  |  | 0.001 |
| no depressive symptoms | 2 (ref) | 75.8 (394) | 86.9 (205) |  |
| mild | 1 | 15.4 (80) | 6.4 (15) |  |
| severe | 0 | 8.8 (46) | 6.8 (16) |  |
| Disease count, M (SD) |  | 2.34 (1.24) | 2.08 (1.16) | 0.005 |
| Medication (total), M (SD) |  | 6.74 (3.93) | 5.54 (3.48) | <0.001 |
| Medication (categorical), % (n) |  |  |  | 0.009 |
| 0-2 | 0 | 13.5 (77) | 21.8 (53) |  |
| 3-4 | 1 | 16.5 (94) | 17.3 (42) |  |
| ≥5 (polypharmacy) | 2 (ref) | 70 (399) | 60.9 (148) |  |
| Non-prescribed medication, % (n) |  |  |  | 0.004 |
| yes | 1 (ref) | 38.9 (222) | 49.8 (121) |  |
| no | 0 | 61.1 (348) | 50.2 (122) |  |
| Arthritis in hand(s) % (n) |  |  |  | <0.001 |
| yes | 1 (ref) | 53 (9.3) | 2.1 (5) |  |
| no | 0 | 517 (90.7) | 97.9 (238) |  |
| Falls in previous year, % (n) |  |  |  | 0.03 |
| none | 2 (ref) | 59.6 (326) | 66.7 (158) |  |
| 1-2 | 1 | 44.0 (241) | 28.7 (68) |  |
| 3 and more | 0 | 28.5 (156) | 4.6 (11) |  |
| Retention, % (n) |  |  |  | <0.001 |
| completed the study | 1 (ref) | 36.3 (207) | 53.1 (129) |  |
| dropped out | 0 | 63.7 (363) | 46.9 (114) |  |

^*^Based on cut-offs of ≤27 (men) and ≤16 (women), or ≤2.5 T-score below sex-specific young adult peak mean [4].

^†^χ^2^ for categorical, Student’s t- and Mann-Whitley U-test for ordinal and non-normally distributed variables.

GDS, Geriatric Depression Scale; GS, grip strength; M, mean; SD, standard deviation; SMMSE, Standardized Mini-Mental State Examination.
