# Supplementary material for: Grip Strength Decline and Its Determinants in the Very Old: Longitudinal Findings from the Newcastle 85+ Study
Source: PLoS One. 2016 Sep 16;11(9):e0163183. doi: 10.1371/journal.pone.0163183 (PMC5026378; doi:10.1371/journal.pone.0163183)
Supplement: S3 Table — (DOCX) [file pone.0163183.s006.docx]

**S3 Table.** Grip strength trajectory estimates^†^ in the survivor sub-cohort (n=343) over 5 year

| Fixed effect^‡^ | Model 1^¶^ |  | Model 2^#^ |  |
| --- | --- | --- | --- | --- |
|  | β (SE) | p | β (SE) | p |
| **GS intercept** | 18.37 (0.42) | <0.001 | 8.58 (1.50) | <0.001 |
| Sex |  |  |  |  |
| men |  |  | 12.16 (0.60) | <0.001 |
| women |  |  | 0 |  |
| Marital status |  |  |  |  |
| single |  |  | 1.97 (0.89) | 0.03 |
| widowed/separated/divorced |  |  | 1.46 (0.57) | 0.01 |
| married (ref) |  |  | 0 |  |
| Physical activity |  |  |  |  |
| high |  |  | 3.98 (0.90) | <0.001 |
| medium |  |  | 2.59 (0.88) | 0.004 |
| low (ref) |  |  | 0 |  |
| Height |  |  | 0.23 (0.06) | <0.001 |
| FFM |  |  | 0.16 (0.05) | <0.001 |
| Disease count |  |  | -0.62 (0.22) | 0.006 |
| Arthritis in hand(s) |  |  |  |  |
| no |  |  | 3.00 (0.93) | 0.001 |
| yes (ref) |  |  | 0 |  |
| **GS decline** |  |  |  |  |
| Time^c^ | -0.34 (0.12) | 0.004 | -0.21 (0.12) | 0.09 |
| Time*Time^c^ | -0.09 (0.22) | <0.001 | -0.08 (0.02) | <0.001 |
| **GS rate of decline (slope)** |  |  |  |  |
| Time*sex |  |  |  |  |
| Time*men |  |  | -0.47 (0.09) | <0.001 |
| Time*women |  |  | 0 |  |

^†^Estimated β coefficients (SE) using GS longitudinal data.

^‡^Fixed effects assed population average change in GS. Fixed effect covariates estimated initial level and trajectory differences in GS as a function of included covariate. Random effects included both intercepts and slopes (linear change). Time in years (continuous) since the baseline interview tested linear change. Time*Time (continuous) represented curvilinear change.

^¶^Model 1 includes a linear and quadratic trend of time.

^#^Model 2 is adjusted for sociodemographic, lifestyle, anthropometry, health-related factors, and time interaction terms. Only significant predictors and interactions at first entry were retained in the model.

FFM, fat-free mass; GS, grip strength; ref, reference.
